# Effect of Combination of Blue and Red Light with Terbinafine on Cell Viability and Reactive Oxygen Species in Human Keratinocytes: Potential Implications for Cutaneous Mycosis

**DOI:** 10.3390/ijms252212145

**Published:** 2024-11-12

**Authors:** Luis Alfonso Pérez González, María Antonia Martínez-Pascual, Elena Toledano-Macías, Rosa Cristina Jara-Laguna, Montserrat Fernández-Guarino, María Luisa Hernández-Bule

**Affiliations:** 1 Dermatology Service, Instituto Ramón y Cajal de Investigación Sanitaria (Irycis), Hospital Ramón y Cajal, Ctra. Colmenar Viejo, km. 9.100, 28034 Madrid, Spain; pg.l.alfonso@gmail.com; 2 Photobiology and Bioelectromagnetic Laboratory, Instituto Ramón y Cajal de Investigación Sanitaria (Irycis), Hospital Ramón y Cajal, Ctra. Colmenar, km. 9.100, 28034 Madrid, Spain; m.antonia.martinez@hrc.es (M.A.M.-P.); elena.toledano@hrc.es (E.T.-M.); rosacristina.jara@salud.madrid.org (R.C.J.-L.)

**Keywords:** onychomycosis, physical therapies, keratinocytes, reactive oxygen species, blue light

## Abstract

Cutaneous mycoses are common infections whose treatment has become more complex due to increasing antifungal resistance and the need for prolonged therapies, hindering patient adherence and increasing the incidence of adverse effects. Consequently, the use of physical therapies, especially photodynamic therapy (PDT), has increased for the treatment of onychomycosis due to its antimicrobial capacity being mediated by the production of reactive oxygen species. This study investigates the in vitro effect of applying blue light (448 nm) or red light (645 nm), alone or together with terbinafine, on the viability of human keratinocytes and the production of reactive oxygen species. The combination of terbinafine and blue light significantly increases ROS production and caspase-3 expression, while red light together with terbinafine increases catalase, superoxide dismutase (SOD) and PPARγ expression, which reduces the amount of ROS in the cultures. The effect of both treatments could be useful in clinical practice to improve the response of cutaneous mycoses to pharmacological treatment, reduce their toxicity and shorten their duration.

## 1. Introduction

Cutaneous mycoses are highly prevalent, affecting up to 20–30% of the general population [1]. Among these, onychomycosis accounts for up to 30% of cases and is associated with various predisposing factors such as occupation, social class, climate, underlying diseases (diabetes, peripheral vascular disease, immunodeficiency and psoriasis), age and genetic factors such as HLA. Although it is usually a mild infection without systemic involvement, it often leads to dermatological consultations due to aesthetic alterations and sometimes functional limitations in physical and occupational activities [2,3,4].

Dermatophytes are the most common cause of onychomycosis, with *Trichophyton rubrum* being the most common etiological agent, responsible for more than half of cases. Yeasts, mainly Candida spp., account for up to 20% of cases [1,5]. Additionally, there is a third group of pathogens causing onychomycosis, non-dermatophytic filamentous fungi, including species such as *Aspergillus* spp., *Fusarium* spp., *Scopulariopsis brevicaulis* and *Onychocola canadensis*, comprising around 10% of onychomycosis cases. Despite their lower frequency, managing these fungi is complex due to their survival in non-keratinized tissues, preference for immunocompromised patients and the high resistance of several species (*Scopulariopsis brevicaulis*, *Fusarium oxysporum* and *Neoscytalidium hyalinum*) to commonly used antifungals, especially terbinafine [6,7].

The epidermis, the outermost layer of the skin, is a physical barrier against pathogens. The disturbance of the epidermal barrier plays a key role in protecting against cutaneous mycoses [8,9,10,11]. The pathophysiology of dermatophyte infection involves adhesion to the stratum corneum, followed by invasion into the underlying sublayers. Adhesion is facilitated by fibrils on fungal spore cell walls that anchor to host keratinocyte membranes, as well as carbohydrate-specific adhesins that recognize mannose and galactose on host cells. Following adhesion, spores germinate, forming hyphae that grow in multiple directions, including deeper into the stratum corneum, which results in the destruction of subungual structures [12]. The cornerstone of onychomycosis treatment is oral antifungals, as topical agents have limited ability to penetrate the nail plate, restricting their use as adjuncts or for superficial infections. However, despite the widespread use of systemic antifungals, therapeutic failure rates can reach 30% in some series. Moreover, due to the need for prolonged treatments to last on average 6 to 12 weeks, the likelihood of adverse effects such as hepatotoxicity or drug interactions is high, often precluding elderly patients, those with liver disease or those on multiple medications from this treatment [13,14,15,16].

According to current guidelines and standard clinical practice, oral terbinafine is the preferred antifungal for onychomycosis treatment [17]. Its main mechanism of action is the inhibition of squalene oxidase; however, it also induces oxidative damage in both fungi and epithelial cells. Resistance to this drug was previously rare, and routine sensitivity studies were not performed. However, in recent years, an increasing number of cases of resistance to this drug in common dermatophytic fungi have been reported. It is important to note that yeasts such as *Candida* spp. and especially non-dermatophytic filamentous fungi generally have a high rate of resistance to terbinafine [18,19,20].

Therefore, due to the growing difficulty in treating cutaneous mycoses with antifungals, studies exploring other treatment modalities have been conducted [21]. Physical therapies have emerged as the primary therapeutic alternative to antifungals [22]. Photodynamic therapy (PDT), laser and high-energy pulsed light are increasingly used in the treatment of cutaneous mycoses, especially onychomycosis, due to their antimicrobial properties and its ability to reduce biofilm formation.

Irradiation with visible wavelengths, both blue (400–480 nm) and red (600–750 nm), can increase the production of reactive oxygen species, exerting an antifungal effect by directly damaging dermatophytes and stimulating the immune system. However, their main limitation lies in the difficulty of penetration of the photosensitizer and light into the nail plate. To address this, standard PDT protocols have been adapted for this new location, using prolonged occlusions or hydrocolloid dressings to improve the penetration of the photosensitizer, increasing the intensity of the administered light or using keratolytics to reduce the thickness of the nail plate [23,24,25].

Given terbinafine’s ability to induce the accumulation of reactive oxygen species, combining PDT or other physical therapies with antifungal treatments could generate a synergistic effect, improving therapeutic response, allowing lower doses of antifungals to reduce toxicity and adverse effects, possibly enabling the treatment of previously ineligible patients [26,27,28,29]. Thus, the aim of the present study was to analyze in vitro the effect of a joint treatment of blue light (448 nm) or red light (645 nm) and terbinafine on the viability and generation of free radicals in a model of human keratinocytes (HaCat line), which are the cell type located at the proximal end of the nail and are the only living part of it.

## 2. Results

To determine whether irradiation with visible light of 448 nm or 645 nm, alone or with terbinafine, can cause cytotoxicity, generation of oxidative stress, apoptosis or the expression of antioxidant enzymes in human keratinocytes, cell viability assays (XTT assay) for ROS production, expression of caspase-3, superoxide dismutase (SOD), catalase, glutathione peroxidase (GPX) and PPARγ were performed.

### 2.1. Cell Viability

In order to study the effect of blue or red light alone or in combination with terbinafine on the cell viability of HaCat, the XTT assay was used. The addition of terbinafine to non-exposed light cultures induced a 33.66 ± 6.70% (*p* < 0.0001) reduction in HaCat cell viability in the XTT assay over the control (non-light exposed and terbinafine-free cultures).

Exposure for two consecutive days to blue light also triggered a reduction of 30.42 ± 1.9% (*p* < 0.001) over the control in HaCat viability. Similarly, in terbinafine and blue light-exposed cultures, viability was reduced by 56.18 ± 2.25% (*p* < 0.0001) over the control.

**Figure 1:**
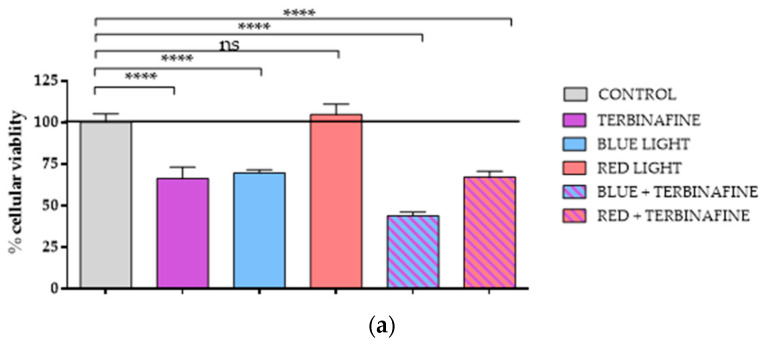

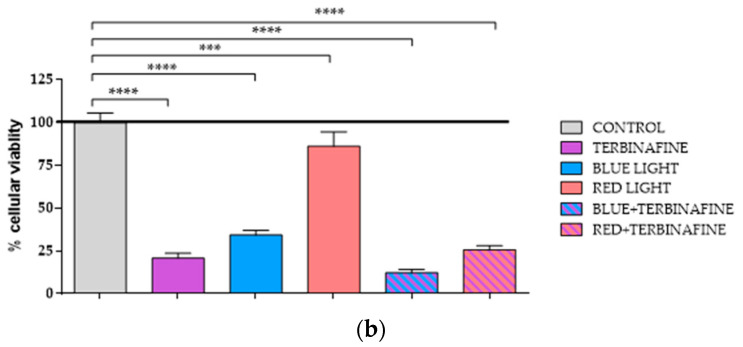
XTT assay. XTT viability assay in HaCaT cells treated with terbinafine (1 µM), blue light (448 nm), red light (654 nm) or the combination of blue light + terbinafine or red light + terbinafine. Cells treated twice on consecutive days. (**a**) XTT assay under standard conditions of culture. (**b**) XTT assay under nutrient-restricted conditions. Mean ± SD; between 5 and 6 experimental replicates. One-way ANOVA; ***: *p* ≤ 0.001; ****: *p* ≤ 0.0001; ns: *p* > 0.05.

Red light did not show changes in cell proliferation after two consecutive days of treatment. The group treated with red light increased by 4.76 ± 6.22% in terms of cell proliferation compared with the control without statistically significant differences (*p* > 0.05). The combination of red light and terbinafine resulted in a 32.89 ± 3.60% decrease in cellular viability compared with the control (*p* < 0.0001); however, differences compared with the group treated solely with terbinafine were not statistically significant (Figure 1a).

Additionally, HaCat cells were incubated in DMEM in the absence of fetal bovine serum (0% FBS). The aim of this experiment was to study in vitro the low nutrient input of nails affected by onychomycosis. In this case, the XTT assay showed that in cultures with a restriction of serum, the addition of terbinafine to non-light exposed cells induced a 79.18 ± 2.87% (*p* < 0.0001) reduction in HaCat viability over the control. The exposure to blue light in nutrient-restricted conditions also decreased over the control by 65.69 ± 2.65% (*p* < 0.0001) in cell viability. The combination of terbinafine and blue light reduced cell viability by up to 87.73 ± 1.98% (*p* < 0.0001) over the control in the XTT assay. In nutrient-restricted conditions, the group treated with red light exhibited a decrease of 13.75 ± 8.4% (*p* < 0.001) in cell proliferation compared with the control. The combination of red light and terbinafine resulted in a 74.34 ± 2.44% (*p* < 0.0001) decrease in cellular viability compared with the control; when comparing the group treated solely with terbinafine and the group treated with red light and terbinafine, no statistically significant differences were found (Figure 1b).

### 2.2. Reactive Oxygen Species (ROS) Production

**Figure 2:**
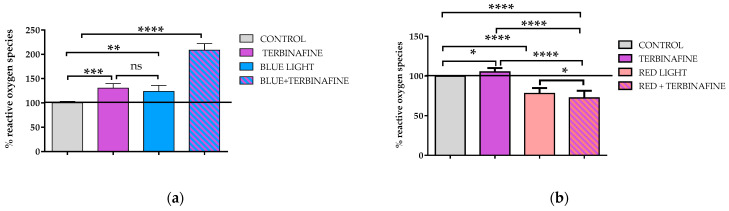

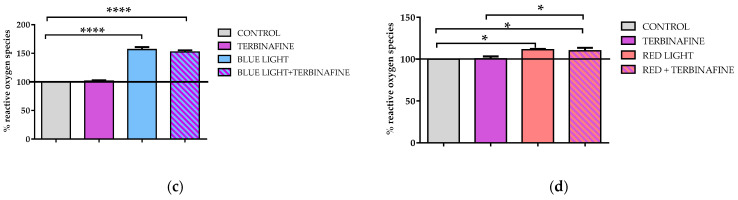
ROS production quantification assay. (**a**) HaCaT treated with terbinafine (1 µM), blue light (448 nm) or the combination of blue light + terbinafine twice on consecutive days. (**b**) HaCaT treated with terbinafine (1 µM), red light (654 nm) or the combination of red light + terbinafine twice on consecutive days. (**c**) HaCaT treated with terbinafine (1 µM), blue light (448 nm) or the combination of blue light + terbinafine once. (**d**) HaCaT treated with terbinafine (1 µM), red light (654 nm) or the combination of red light + terbinafine once. Mean ± SD; *n* = 4 experimental replicates. One-way ANOVA: * *p* < 0.05; ** *p* ≤ 0.01; *** *p* ≤ 0.001; **** *p* ≤ 0.0001.

To determinate the ROS production induced by blue or red light, quantitative immunofluorescence assays were performed to assess the amount of fluorescent dye dichlorofluorescein as a measure of the level of ROS. Quantification of ROS in cells treated for two consecutive days with terbinafine alone showed an increase of 30 ± 9.2% (*p* < 0.001) in the cultures. Furthermore, cultures exposed only to blue light twice showed an increase of 23 ± 12.5% (*p* < 0.001) in ROS production, while the combination of blue light and terbinafine increased their production by up to 108% (SD 13.4% *p* < 0.001) compared with the control. (Figure 2a). On the other hand, two consecutive days of exposition to red light without terbinafine provoked a decreased 21.5 ± 3.4% (*p* < 0.01) in ROS production. When the cultures were exposed to red light in the presence of terbinafine, an even greater decrease in ROS was observed compared with the control than when they were exposed to red light alone (27.6 ± 4.5%). This difference was statistically significant (*p* < 0.05) (Figure 2b).

Additionally, the effect on ROS production of a single exposure to blue or red light was analyzed. For this purpose, the same doses of light and terbinafine were used as in the experiments mentioned above, but the amount of ROS produced was analyzed immediately after a single exposure to light. The exposure one time to blue light without terbinafine provoked a significant increase in ROS (56.56 ± 4.24% over the control, *p* < 0.0001). Co-treatment with terbinafine and a single exposure to blue light also significantly increased ROS production (52.29 ± 2.92% over the control) (Figure 2c). Also, one exposure to red light caused a significant increase in reactive oxygen species both, in the presence (11.36 ± 0.80%; *p* < 0.005) or absence of terbinafine (10.08 ± 3.49%, *p* < 0.05) (Figure 2d).

### 2.3. Expression of Antioxidant Enzymes Superoxide Dismutase (SOD), Catalase, Glutathione Peroxidase (GPX) and PPARγ in HaCat Exposed to Blue Light

**Figure 3:**
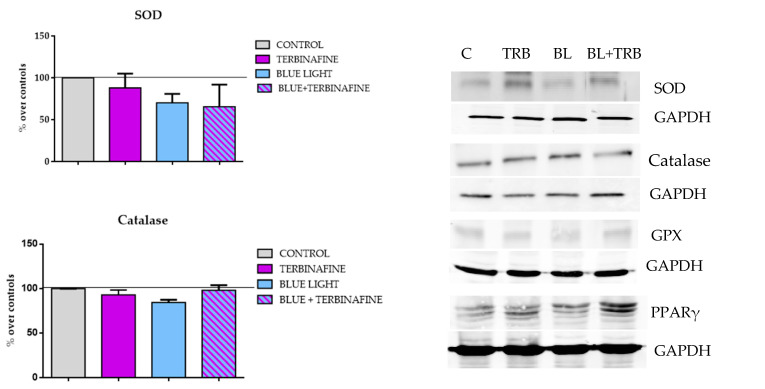

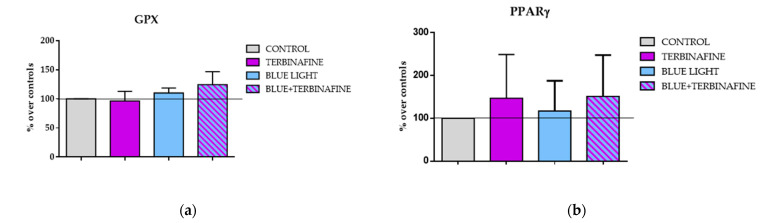
Superoxide dismutase (SOD), catalase, glutathione peroxidase (GPX) and PPARγ expression. Cells were treated twice on consecutive days. (**a**) Densitometry values for SOD, catalase, GPX and PPARγ expressions. Data normalized over the corresponding sham treatment controls (solid line represents the control group 100%). Means ± SD of the protein/GAPDH ratios of at least four experimental repeats per protein. One-way ANOVA. (**b**) Representative blots (30 g of protein/lane). GAPDH was used as the loading control. Sham treatment (C), terbinafine (TRB), blue light (BL), blue light + terbinafine (BL + TRB).

To establish the implication of antioxidant pathways in the response to terbinafine and/or blue or red light treatment, the expression of SOD, GSH-Px, catalase and PPARγ was analyzed. The results showed no significant changes with respect to the control when the cells were exposed to two doses of blue light, terbinafine or joint treatment for any of the enzymes analyzed (Figure 3).

### 2.4. Expression of Antioxidant Enzymes Superoxide Dismutase (SOD), Catalase, Glutathione Peroxidase (GPX) and PPARγ in HaCat Exposed to Red Light

**Figure 4:**
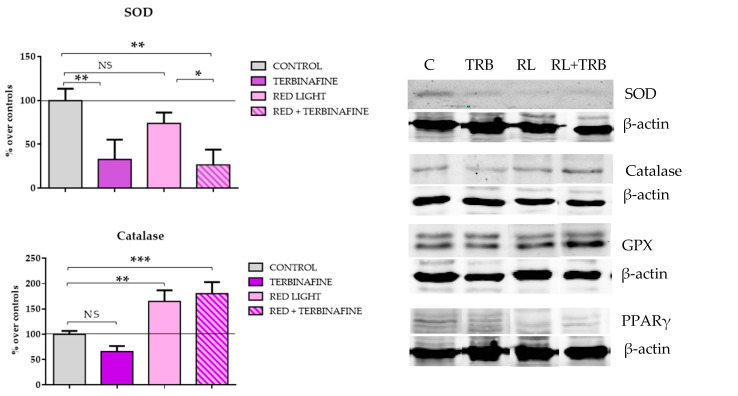

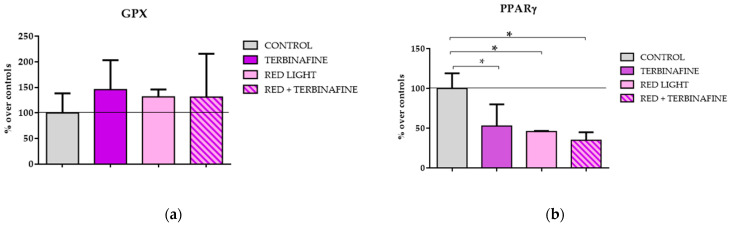
Superoxide dismutase (SOD), catalase, glutathione peroxidase (GPX) and PPARγ expression. Cells were treated twice on consecutive days. (**a**) Densitometry values for SOD, catalase, GPX and PPARγ expressions. Data normalized over the corresponding sham treatment controls (solid line represents the control group 100%). Means ± SD of the protein/β-actin ratios of at least four experimental repeats per protein. One-way ANOVA: NS: *p* ≥ 0.05; * *p* ≤ 0.05; ** *p* ≤ 0.01; *** *p* ≤ 0.001. (**b**) Representative blots (30 g of protein/lane). β-actin were used as the loading control. Sham treatment (C), terbinafine (TRB), red light (RL), red light + terbinafine (RL + TRB).

The expression of these enzymes was also analyzed in the presence or absence of red light and with or without treatment with terbinafine. After two doses of red light, a significant decrease in SOD expression in cultures treated with terbinafine (26.5 ± 17.47% of the control, *p* < 0.01 **) or red light and terbinafine (32.64 ± 22.62% of the control, *p* < 0.01 **) was detected (Figure 4a,b). No significant changes were observed in cultures exposed only to red light.

On the other hand, treatment with red light alone caused a significant increase in the expression of catalase (64.87 ± 22.01%; *p* < 0.01 ** over the control). In cultures exposed to red light and treated with terbinafine, this enzyme was also significantly increased (80.15 ± 23.02%; *p* < 0.001 ***). No changes were observed in GPX expression (Figure 4a,b).

Regarding PPARγ, treatment with terbinafine alone caused a significant decrease in its expression (52.83 ± 27.05%; *p* < 0.05). PPARγ expression also decreased significantly in cells exposed to red light alone (45.93 ± 0.69%; *p* < 0.05) and in those exposed to both treatments (34.88 ± 9.99% *p* < 0.05) (Figure 4a,b).

### 2.5. Caspase-3 Expression

**Figure 5:**
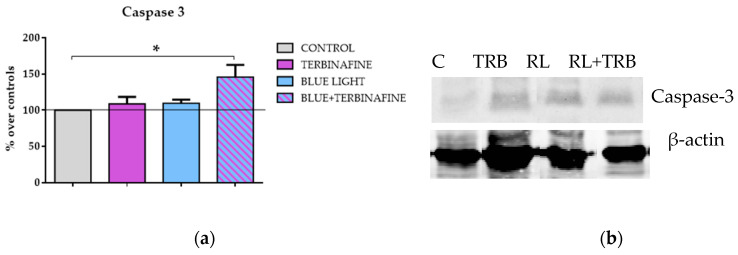

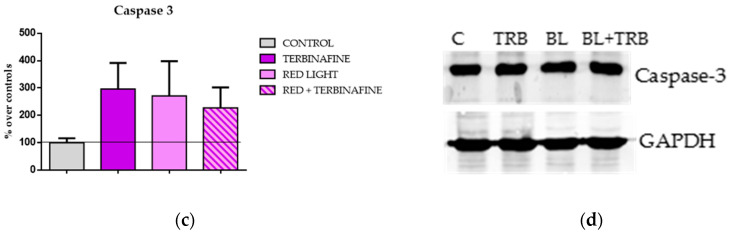
Caspase-3 expression. Cells were treated twice on consecutive days. (**a**) Densitometry values for caspase-3 expressions. Data normalized over the corresponding sham treatment controls (solid line represents the control group 100%). Blue light. Means ± SD of the protein/GAPDH ratios of at least four experimental repeats per protein. One-way ANOVA: * *p* < 0.05. (**b**) Sham treatment (C), terbinafine (TRB), blue light (BL), blue light + terbinafine (BL + TRB). (**c**) Densitometry values for caspase-3 expressions. Red light. Data normalized over the corresponding sham treatment controls. Means ± SD of the protein/GAPDH ratios of at least four experimental repeats per protein. One-way ANOVA. (**d**) Representative blots. GAPDH or β-actin were used as the loading control. Sham treatment (C), terbinafine (TRB), red light (RL), red light + terbinafine (RL + TRB).

The antioxidant system comprises both antioxidant enzymes and non-enzymatic antioxidants capable of preventing oxidative damage in human skin [30]. Therefore, the expression of relevant proteins in the antioxidative defense, such as caspase-3, was also analyzed. The interest in the study of this protein is also related to its involvement in the response to blue light treatments in different cell types [31,32]. An immunoblot analysis of caspase-3 expression revealed a significant increase in the expression of this protein (24.44 ± 31.6%; *p* < 0.05 *) when cells were treated with light and terbinafine. No changes were observed when samples were treated with light or terbinafine alone. Caspase-3 expression was also analyzed in HaCat treated with red light in the presence or absence of terbinafine. Treatments alone or in combination induced increases in caspase expression, but the results were not statistically significant (Figure 5).

## 3. Discussion

The use of physical therapies in the treatment of onychomycosis has increased in recent years due to rising therapeutic failures with oral antifungal agents. The growing number of publications in recent years reveals promising results, demonstrating the efficacy and safety of these therapies [33,34,35,36,37]. However, there is a significant difficulty in reaching firm conclusions due to the significant heterogeneity among studies. A recent systematic review and meta-analysis of PDT use in dermatophytosis suggest its effectiveness, with cure rates ranging from 17% to 80% [23,25]. Variations in efficacy depend on the photosensitizer used and number of sessions, and concurrent treatments were detected. Similarly, systematic reviews and meta-analyses evaluating the efficacy of pulsed dye laser, Nd:YAG and CO_2_ in onychomycosis have reported similar results to PDT, achieving cure rates of 63% to 74% [38,39]. In addition to their antimicrobial effect, light- and laser-based therapies appear to have the ability to disrupt biofilm formation [40]. In this sense, Vila et al. [41] demonstrated that Nd:YAG laser and intense pulsed light (IPL) treatment at 420 nm can reduce biofilm formation by approximately 50% in fungal cultures.

The production, regulation and response of reactive oxygen species are a central axis of host–pathogen interactions [42]. Both hosts and fungi produce reactive oxygen species, utilize conserved mechanisms to metabolize these radicals and leverage them in their local environment to mediate defense mechanisms [28,43]. In the skin, to prevent damage caused by excessive levels of ROS and regulate epidermal homeostasis, cells activate their endogenous defense system and exert their antioxidant function. The enzymes superoxide dismutase (SOD), catalase and glutathione peroxidase (GPX) act as ROS detoxifiers. However, the sustained production of ROS and other highly reactive species, not sufficiently removed by antioxidant enzymes, can cause significant damage to living cells. Thus, reactive oxygen species can react with polyunsaturated fatty acids in cell membranes, nucleotides and sulfhydryl bonds in proteins and have been associated with tissue damage in fungal infections [44,45,46,47,48,49].

Blue and red light at certain fluences are known to be capable of generating oxidative stress and ROS. If the damage is too great, it can lead to necrotic or apoptotic cell death. This light-generated oxidative effect has been used in PDT to directly damage dermatophytes. On the other hand, a strong association has been described between the lethality of antifungal drugs and the formation of ROS, which would provoke apoptosis or necrosis in the fungi causing the pathology. Although this oxidative effect on the viability of the fungus causing onychomycosis has not been analyzed in the present study, the mechanism has been widely described in the literature [28]. However, evidence on the combined use of PDT with other treatments is scarce and mainly limited to small series of patients receiving heterogeneous treatments, and there are no experimental studies examining the molecular effect of combining light treatments with antifungals, limiting the evidence to isolated case reports [50,51,52].

**Figure 6:**
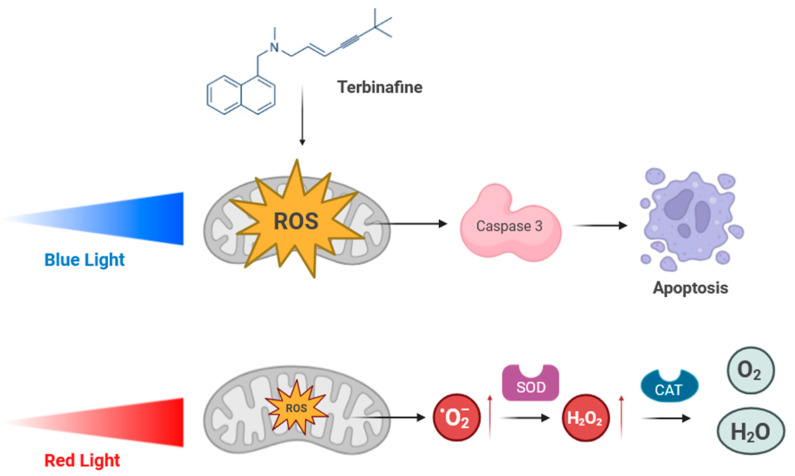
Synergistic effect of terbinafine and blue light or terbinafine and red light. Proposed mechanism of action. Treatment with blue light or red light with terbinafine causes increases in ROS in keratinocytes. The combination of blue light and terbinafine significantly increases free radicals in the culture, while the combination with red light only increases them weakly. This effect would be due to an activation of different cellular response mechanisms depending on the wavelength applied. Thus, the combination of blue light and terbinafine would act on the mitochondria of keratinocytes, causing an increase in the proportion of ROS and, consequently, the activation of the proapoptotic enzyme caspase-3. Such activation, together with the inhibition of fungal catalase by blue light, would help promote the elimination of dermatophytes from the tissue. On the other hand, the slight increase in free radicals after treatment with red light and terbinafine triggers the activation of tissue catalase, leading to a reduction in ROS and thus increasing resistance to oxidative stress. Red arrows: increases in the amount of free radicals.

In the present study, keratinocyte cells were used as an experimental model of the cells that generate the nail and, consequently, as the main cell type affected in onychomycosis. The results of our study demonstrate that blue light and terbinafine decrease the viability of keratinocyte cultures. Furthermore, the combination of blue light therapy and terbinafine increases the cytotoxic effect of both therapies. This cytotoxicity is probably due to the significant increase in the production of reactive oxygen species in the cultures, although no significant overexpression of any of the antioxidant enzymes was detected after pharmacological and/or blue light treatment. On the contrary, caspase-3, involved in the apoptosis process, increased its expression when the treatment combined blue light and terbinafine. This synergistic effect is intensified under in vitro conditions of nutrient restriction simulating onychomycosis, thus mimicking the limited nutrient and oxygen supply due to the inadequate acral circulation characteristic of this pathology. Under the same nutrient-restrictive conditions, red light also slightly decreased cell viability compared with the control. This effect is likely due to nutrient-restricted cultures being more sensitive to external stimuli such as red light than under normal culture conditions, where it would not affect viability. Moreover, when both chemical and light treatments were applied together in these SFB-depleted cultures, the combined effect was much greater than that observed in cultures grown under standard conditions and exposed to both treatments. Thus, cell viability results correlate with reactive oxygen species quantification. Mitochondria have been suggested by other authors as a potential target for the action of both terbinafine and blue light [27,53]. The effect of both agents on mitochondria would be consistent with the observed results of a decrease in viability, together with an increase in free radicals and an increase in caspase-3 expression (Figure 6).

In contrast, red light did not affect cell viability and significantly reduced ROS production. When red light was combined with terbinafine, a decrease in cell viability did occur, which would be solely attributable to the cytotoxic effect of terbinafine. This red light treatment also significantly increased the antioxidant enzyme catalase, while red light combined with terbinafine decreased SOD expression. Surprisingly, a single exposure to red light did cause a moderate but statistically significant increase in ROS in the culture. This increase in ROS was even greater when combined with terbinafine. Given its lower energy capacity and greater penetration ability, red light is likely capable of inducing a slight increase in cellular oxidative stress, insufficient to cause necrosis or apoptosis but capable of inducing a higher expression of catalase, thereby increasing the cells’ global resistance to oxidative stress (Figure 6).

Different studies have shown that PPARγ is involved in the response to oxidative stress. PPARγ can directly modulate the expression of several antioxidant and pro-oxidant genes in response to oxidative stress. Thus, it has been described that mouse, human and rat catalase are transcriptionally regulated by PPARγ [54]. In the present study, exposure to blue light did not cause significant changes in these proteins, but when cells were exposed to red light alone or in combination with terbinafine, PPARγ expression decreased and catalase expression increased in the cultures. These responses would be indicative of an effect of red light on PPARγ-mediated oxidative stress enzymes.

On the other hand, increased hydrogen peroxide (H_2_O_2_) generation plays an important role in eliminating pathogenic microorganisms [46,55,56]. It was described that catalase residing inside bacteria could be effectively inactivated by blue light, subsequently rendering the pathogens extremely vulnerable to H_2_O_2_ and H_2_O_2_-producing agents [57]. Our experiments demonstrated that the terbinafine and blue light, alone or in combination, induced in HaCat increased ROS production. Recent studies have shown that 405 nm blue light can effectively inactivate catalase in a variety of susceptible and resistant dermatophytes of the genus *Trichophyton*. Light-treated dermatophytes showed increased sensitivity to reactive oxygen species (ROS)-producing agents, which improved the performance of antimicrobial agents such as H_2_O_2_ and amphotericin B. Light-induced inactivation of catalase inhibited the formation and polarized growth of dermatophytes, suppressing biomass formation [58]. Thus, excess ROS production in keratinocytes together with the inhibition of fungal catalase by blue light would help promote the elimination of dermatophytes from the tissue. However, the combined effect of light and pharmacological therapies such as terbinafine on the fungus remains to be studied. Therefore, it will be necessary to carry out in vitro microbiological studies to confirm whether the increase in reactive oxygen species is also accompanied by an increase in the antifungal effect, as well as trials in patients to reveal the clinical utility of this combination of treatments investigated here.

One aspect of the study that could seem controversial is the safety of blue light. Some studies have shown that high-energy visible light (HEVL) could potentially induce DNA mutations through an indirect mechanism related to oxidative stress, similar to that caused by ultraviolet radiation, given the proximity of high-energy blue light (400–415 nm) to UVA radiation in the visible spectrum [59]. However, the blue light used in our study corresponds to a wavelength (448 nm) further from UVA than HEVL, and the exposure duration used in the treatment is much shorter than what has been described as causing this potential indirect mutagenic effect [60].

In conclusion, the effect of exposure to blue light and terbinafine would increase the proportion of ROS in keratinocytes, which would act by eliminating pathogenic microorganisms. On the other hand, treatment with red light would cause a slight increase in free radicals, leading to the activation of catalase, which would trigger a reduction in the amount of free radicals present in the tissue. This synergistic effect of both light therapies (red and blue) and pharmacological (terbinafine), if it occurs in nails affected by mycosis, could be useful in clinical practice to improve its treatment, either by improving cure rates compared with standard treatment with terbinafine or by using this combination to shorten the duration of treatment with terbinafine, thus reducing possible toxicities and drug interactions.

## 4. Materials and Methods

Cell culture: Human keratinocytes (HaCaT cell line, CLS Cell Lines Service, 300493, Eppelheim, Germany) were seeded in medium composed of high-glucose DMEM (Biowhittaker, Lonza, Verviers, Belgium) supplemented with 10% inactivated fetal bovine serum (Gibco, Waltham, MA, USA), 2 mM L-glutamine, 100 U/mL penicillin, 100 U/mL streptomycin and 0.25 μg/mL of amphotericin B (Gibco) and maintained in a 5% CO_2_ atmosphere at a temperature of 37 °C inside CO_2_ incubators (Thermo Fisher Scientific, Waltham, MA, USA). The cells were sub-cultured once a week, and the culture medium replaced each 3 days.

For experiments, HaCaT were seeded in 24- or 96-well multi-well plates (Corning Costar TC-Treated Multiple Well Plates, New York, NY, USA) and incubated with DMEM supplemented with 10% fetal bovine serum, 2 mM L-glutamine, 100 U/mL penicillin, 100 U/mL streptomycin and 0.25 μg/mL of amphotericin B. Furthermore, in a series of experiments, HaCat cells were incubated in DMEM in the absence of serum (0% FBS) but in the presence of the rest of the culture medium components described above. Cells were growth for 2 days in a 5% CO_2_ atmosphere at a temperature of 37 °C to provide adherence to the bottom of the plate. Only 12 wells were seeded to exposure with the light exposition device. Thirty minutes before the light exposition, the culture medium was changed to a non-colored maintenance culture medium to avoid interference with light treatments and colorimetric measurements [61].

Terbinafine incubation: Terbinafine drug was added to half of the wells immediately before exposure to light. The terbinafine dosage used in this study was 1 µM (terbinafine hydrochloride, Merck, 78628-80-5, Darmstadt, Germany). This dosage was the same used in other publications that previously exposed keratinocytes to this drug [32,62].

**Figure 7:**
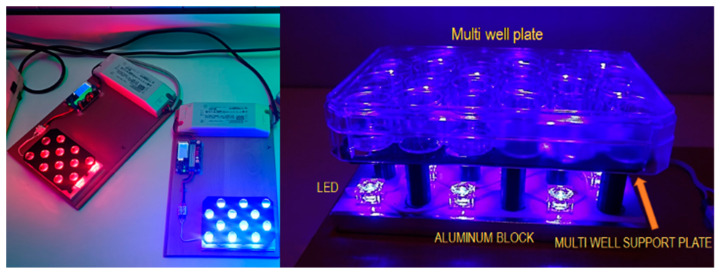
Exposure system (see text).

Blue or red light exposure: The light exposure was performed with an original 3D impression specific device that emits 448 nm blue light and 645 nm red light. The device was equipped with 12 blue LEDs (Lumileds LUXEON Rebel color Royal Blue 448 nm LXML-PR01-0500) and 12 red LEDs (Lumileds LUXEON Rebel Color Red 645 nm LXM2-PD01-005, Schiphol, The Netherlands) [61]. Our exposure device allows for controlled treatments and includes a focusing lens that ensures that the exposure of the culture is homogeneous (Figure 7).

The light treatments consisted of one or two irradiation sessions of 52 mW/cm^2^ (blue or red light) for 20 min, applied once or every 24 h for two consecutive days. The experiment was controlled by a multi-well plate cultured in identical conditions but in the dark.

Cell proliferation XTT assay: HaCaT cultures were seeded at densities of 4500 cells/cm^2^ and incubated for 3 days [63]. To determinate the effects of terbinafine and light exposition in HaCaT, proliferation and viability XTT assays (cell proliferation assay Roche, Schweiz, Switzerland) were performed. Cell Proliferation Kit XTT employs 2, 3-Bis-(2-methoxy 4-nitro-5-sulfophenyl)-2H-tetrazolium5-carboxanilide salt (XTT). Mitochondria are only capable of reducing XTT to form an orange-colored water-soluble dye in living cells. Therefore, the concentration of the dye is proportional to the number of metabolically active cells. XTT assays were carried out 24 h after the second light exposure.

Reactive oxygen species (ROS) assay: For the ROS assay, cells were seeded at a density of 4500 cells/cm^2^ and incubated for 3 days. To quantify the ROS accumulation in the cultures due to terbinafine and light exposition (blue or red), an immunofluorescence assay was performed. ROS assays were carried out immediately after first or second exposure to minimize their degradation. ROS production was assessed by the quantification of the intracellular oxidative transformation of the oxidation-sensitive probe DCFH-DA into the fluorescent dye dichlorofluorescein (DCF). Then, HaCat cultures were incubated with the fluorescent probe 2′7-Dichloro dihydrofluorescein diacetate (5 μM DCFH-DA, Sigma-Aldrich, San Louis, MO, USA) for the dark at 37 °C and 5% CO_2_ during 30 min. They were then read using a TECAN plate reader (TECAN SpectraFluor, Gödrig, Austria) at a λexc 490 nm, λemi 535 nm wavelength [64].

Inmunoblot assay for superoxide dismutase (SOD), glutathione peroxidase (GPX), catalase, PPARγ and caspase-3: HaCaT cells were plated at a 6800 cell/cm^2^ density and incubated for 4 days. The immunoblot procedure has been described in detail elsewhere [65,66]. Briefly, the total cell lysate was obtained by lysing cells in RIPA buffer (Thermo Fisher Scientific) containing protease/phosphate inhibitor cocktail (Thermo Fisher Scientific). Protein content was determined by a BCA protein assay kit (Pierce; Thermo Fisher Scientific). Equal protein volumes (50 μg) were separated in 10% sodium dodecyl sulphate-polyacrylamide gel and electrophoretically transferred to nitrocellulose membrane (Amersham, Buckinghamshire, UK). The membranes were incubated at 4 °C overnight in rabbit polyclonal anti-catalase (1:1000, Invitrogen, Waltham, MA, USA), rabbit polyclonal anti-glutathione peroxidase 2 antibody (1:1000, Abcam, Cambridge, UK), mouse monoclonal anti-PPAR γ (Santa Cruz Biotechnology, Heidelberg, Germany), rabbit monoclonal cleaved caspase-3 antibody (1:1000; Cell Signaling, Danvers, MA, USA) and monoclonal rabbit anti-superoxide dismutase 1 (Abcam, Ab 51254). Monoclonal mouse anti-GAPDH (1:1000, Santa Cruz Biotechnology) or mouse monoclonal anti-β-actin (Sigma-Aldrich) were used as the loading controls. The membranes were incubated for one hour at room temperature, with anti-rabbit IgG conjugated to IRdye 800 CW (1:10,000, LI-COR Biosciences, Lincoln, NE, USA) and with anti-mouse IgG conjugated to IRdye 680 LT (1:15,000, LI-COR Biosciences) or anti-mouse IgG horseradish peroxidase-conjugated antibody (1:10,000 NA931; GE Heathcare, Hatfield, UK) and with anti-rabbit IgG horseradish peroxidase-conjugated antibody (1:5000 NA934; GE Heathcare). For the detection and visualization of the immunoreactive bands, the enhanced detection kit ECL (RPN2132; GE Healthcare; Cytiva, Marlborough, MA, USA) was used. The membranes were scanned with a LI-COR Odyssey scanner (LI-COR Biosciences) or with a Bio-Rad imaging system (Hercules, CA, USA). The obtained bands were densitometrically evaluated (PDI Quantity One 4.5.2 software, BioRad, Hercules, CA, USA). At least 3 experimental replicates were conducted per protein. All values were normalized over the loading control.

Statistical analysis: At least three independent replicates were conducted per experiment. Data were normalized and expressed as the means ± standard deviation (SD) of at least three independent experimental runs. A one-way ANOVA with Tukey’s post-test or a two-tailed Student’s *t*-test was applied using GraphPad Prism 6.01 software (GraphPad Software, San Diego, CA, USA) when comparing multiple or two experimental groups, respectively. Differences of *p* < 0.05 were considered statistically significant.

## Data Availability

The data presented in this study are available on request from the corresponding author.
